# Topics of the Month

**Published:** 1897-04

**Authors:** 


### TOPICS OF THE MONTH.

THERE seems to be no end to the perplexities that arise concerning the state medical practice act. The medical profession of the state of New York struggled from 1883 to 1890 to establish a separate state examination for license to practise medicine. Finally, a law was approved, June 5, 1890, to take effect September 1, 1891, providing for a state medical examining board to be appointed by the regents of the University of the State of New York, on the nomination of the three state medical societies representing the several so-called systems of medicine recognised by law. This plan, that seemed somewhat complicated at first, has proven to be a practical solution of what appeared to many to be at first a difficult problem.

The law, however, had hardly taken effect when malcontents and persons with grievances, real or fancied, began to move for amendments. First came the students, who, not satisfied with fifteen months' notice to prepare for the new conditions, prayed for exemption from the operations of the law for another year. The legislature granted this petition, under which 913 medical students, alarmed at the new order of things, obtained exemption from the provisions of the law. A similar attempt was made the following year, but this was defeated, and, in so far as students were concerned, the principle of separate examination for license was definitely settled.

---

Meanwhile, some of the colleges, emboldened, probably, by the success in the first instance of the students, began to demand modifications of the law that should grant either individual or general concessions in its application. These, too, were met and defeated, year by year, in the legislative halls in their attempt to weaken the system and lower the standard. The most conspicuous example occurred about a year ago. Every available influence was then brought to bear to insure success, and when failure came it was hoped and believed by the friends of higher medical education that the struggle was over, that the discontented elements were routed and that the law henceforth would be permitted to work out its own beneficial results in peaceful serenity.

---

Now, however, when all is working smoothly and well, comes the New York State Medical Association, or at least a few disturbing members in that body, (for we are advised that there is not unanimity, nor is the effort being taken as the official act of the association,) asking to be recognised in the personnel of the state examining board to the extent that the association be allotted one-half of the examiners. The men who have been most conspicuous in presenting and urging this bill, which we print in full on another page, are the very ones who have opposed the law from start to finish; first, during the initial struggles pertaining to the passage of the bill through the legislature, and next, in various attempts to modify, weaken or emasculate the law through unfriendly amendments proposed from time to time since its passage.

---

This latest attempt on a part of the members of the New York State Medical Association to disturb the professional peace and quiet that has prevailed for some time past was cunningly planned. Dr. E. D. Ferguson, of Troy, is, it is stated, Governor Black's physician, and it is also given out, on what authority we know not, that should the bill pass the legislature the governor will surely sign it. It is further stated that Dr. Ferguson, who is secretary of the New York State Medical Association, gave a dinner not long ago, at which Senator Ellsworth, temporary president of the senate, and Mr. Nixon, of Chautauqua, leader of the republican side in the assembly, were present. Next day, by a strange juxtaposition of events, bills were simultaneously introduced in the senate and assembly like the copy on another page. Hence, it has been asserted, probably without any authority in fact, that not only is the governor's sanction given to this measure, but that it has the weighty influence of the leaders of both houses of the legislature. We do not believe, however, that either of these prominent men care a pin's fee for such small legislation and that this adroit attempt to couple their names with it will do more harm than good to the measure under these circumstances. The governor is one of the regents, and he would hardly care to be quoted as endeavoring to promote a measure that might lead to embarrassment.

---

At the request of Dr. A. Walter Suiter, chairman of the Legislative Committee of the Medical Society of the State of New York, the assembly bill was transferred from the Committee of Ways and Means, of which Mr. Nixon is chairman, to that on Public Health, of which Dr. Murphy is chairman. On Thursday, March 18, 1897, a hearing on the bill was had before the Committee on Judiciary in the Senate and the Public Health Committee of the house sitting in joint session. Senator Lexow presided and Dr. Murphy acted as vice-chairman. The friends of the measure were out in full force, the country contiguous to Albany yielding a liberal attendance in response to a confidential circular from Dr. Ferguson, who acted as party-whip with the title of "chairman of committee." The hearing lasted nearly two hours, and was listened to with dignified patience by the chairman and all the members of both committees. Senator Lexow announced that two speakers from each side would be heard and that the opposition should begin the debate. Drs. A. Walter Suiter, of Herkimer; Edward Clark, Buffalo; Frank Van Fleet, of New York; J. M. Winfield, of Brooklyn, and Seneca D. Powell, of New York, presented the argument for the Medical Society of the State of New York. Drs. Ferguson, Didama and Truax were the chief spokesmen for the State Medical Association.

---

The arguments may be summarised as follows: The Medical Society of the State of New York contended that since 1806 it had been the representative body of the medical profession, made such by statutory law, and so acknowledged by all the people ; that it is a delegate body in which the several county medical societies are represented by delegates equal in number to the members of assembly ; that it is a legislative body acting in such capacity for about 6,000 physicians, members of the county medical societies ; that if any counties are unrepresented it is by their own volition ; that practically all physicians in the state exercise, or ought to exercise, a voice in its proceedings ; that in its representative capacity it is required by law to nominate to the regents names from which the latter may select the state board of medical examiners ; that it was chiefly instrumental in obtaining the passage of the law creating this reform, and the state could only recognise the three several state medical societies that are created by law in making up the three several state medical examining boards.

It conceded that the New York State Medical Association was a voluntary body, which, even if incorporated, was not created by statutory law, hence not a part of the legal medical organisation of the state ; that it consisted of between 700 and 800 members, most of whom joined by urgent solicitation on the part of drummers ; that all but 296 of this number were members of the county medical societies, and so exercised a voice in the selection of the board of state medical examiners ; that it is controlled in large part by men who seceded from the medical society of the state of New York because the latter, in 1884, abrogated the code of ethics ; that it is an association organised upon a dogma, and fed by a triviality; that its members or some of them have opposed the state medical practice act and have been active in endeavoring to secure amendments to it from time to time that would tend to weaken its force or nullify its influence, and that for these and other reasons it ought not to be allowed representation in its separate capacity on the state board of medical examiners.

---

The New York State Medical Association contended that it was a body loyal to the code of ethics of the American Medical Association, entitled to representation in that body ; that the Medical Society of the State of New York was not entitled to representation in the American Medical Association ; that a former president of the Medical Society of the State of New York had been expelled from the American Medical Association because he remained loyal to the medical society of his own state ; that it is the great apostle of the code of ethics, and that the Medical Society of the State of

New York is a proselyte; that a majority of the members of the county societies are old-codeites; and that for these and other similar reasons it should be given one-half of the emoluments growing out of the organisation and successful management of the State Medical Examining Board, that has been created, fostered and matured under the kindly care and influence of the Medical Society of the State of New York.

---

During the hearing, members of the committee carefully and intelligently interrogated a number of the speakers, thus manifesting an interest in the controversy and a desire to arrive at the precise facts in the case. We have been careful to enumerate only such essential features as actually took place, leaving inference and conjecture as to the real merits of the case to our readers. We have only this remark in explanation to make—namely, that the president of the Medical Society of the State of New York above referred to was not expelled from the American Medical Association, but that he was deposed from the board of trustees of that body, not because of unprofessional conduct, not because of any violation of any of the canons of ethical propriety, but solely because he was a member of the Medical Society of the State of New York and had had the temerity at one time to accept its presidency.

We challenge no man's views in regard to the so-called code of ethics; we believe in the exercise of the greatest liberality on this and kindred subjects relating to the autonomy of the profession wherein honest men may honestly differ; we expect the same liberal treatment at the hands of our friends who hold views different from our own on this comparatively unimportant subject.

---

We never introduce a discussion of the code into the columns of the JOURNAL, regarding it as a subject long since disposed of in this state, and believing that a medical magazine should fill its pages with material of scientific and general professional interest, rather than with the discussion of controversial matters that seldom lead to any satisfactory result. In the present instance we have merely chronicled as journalists the circumstances connected with this surprising change of front on the part of the state association, whereby it now seeks to become a part and parcel of the medical examining system, in which homeopaths, eclectics and nonsectarian physicians are all equal before the law and have share and share alike in the reforms and improvements that are developing.

The New York Physicians' Mutual Aid Association pays its death claims promptly. This fact has recently been exemplified in this city in the payment of $1,000 to Mrs. Dagenais, on March 17th, whose husband, Dr. A. Dagenais, died March 4, 1897—less than two weeks intervening between the death and the date of the payment of the claim.

Membership in the association is limited to physicians residing in this state. Many prominent physicians of Buffalo are members, and it is desirable that the local membership should be still further increased. Dr. S. A. Dunham, 180 West Chippewa street, is the medical examiner and will gladly furnish any information to physicians who may desire to join the association.

